# Supplementary material for: Soil bacterial diversity mediated by microscale aqueous-phase processes across biomes
Source: Nat Commun. 2020 Jan 8;11:116. doi: 10.1038/s41467-019-13966-w (PMC6949233; doi:10.1038/s41467-019-13966-w)
Supplement: Supplementary file 1 — Supplementary Information [file 41467_2019_13966_MOESM1_ESM.pdf]

Supplementary Information

Soil bacterial diversity mediated by microscale aqueous-phase processes across biomes

Authors

Samuel Bickel* and Dani Or

*Correspondence to: SB (samuel.bickel@usys.ethz.ch)

Supplementary Table 1 | Sources of global data and variables utilized in this study. For global maps, the data has been harmonized to a common grid of 0.1°x0.1° (≈ 11 km) determined by the MSWEP dataset.

Name	Resolution	Record	Variables	References
MSWEP v2.1	0.1°, 3-hourly	1979-2016	mean annual precipitiation, mean consecutive dry days	[1]
WorldClim v2	5 arcmin, monthly climatic	1970-2000	mean annual temperature, mean solar irradiance	[2]
SoilGrids	250m & 10km, 7 soil layers	NA	soil texture (sand, silt, clay), bulk density, pH	[3]
MODIS17	1km, annual	2000-2015	mean annual net primary production	[4]

Supplementary Table 2 | Number of samples for groups of climatic water contents (m^3 m^{-3}) in the diversity datasets. Datasets of bacterial diversity (Earth Microbiome Project - EMP[5], Delgado-Baquerizo *et al.* - DEL[6]) were grouped by climatic water contents (EMP additionally by soil depth; Top: <25cm, Sub: ≥25cm). The numbers of samples per group are reported.

Study	Climatic water content class	Soil layer	Number of samples
DEL	(0.0, 0.05]	-	4
DEL	(0.05, 0.1]	-	6
DEL	(0.1, 0.15]	-	28
DEL	(0.15, 0.2]	-	104
DEL	(0.2, 0.25]	-	71
DEL	(0.25, 0.3]	-	23
DEL	(0.3, 0.35]	-	1
EMP	(0.0, 0.05]	Top	3
EMP	(0.05, 0.1]	Top	75
EMP	(0.1, 0.15]	Top	93
EMP	(0.15, 0.2]	Top	253
EMP		Sub	12
EMP	(0.2, 0.25]	Top	1594
EMP		Sub	44
EMP	(0.25, 0.3]	Top	562
EMP		Sub	93
EMP	(0.3, 0.35]	Top	28
EMP		Sub	11

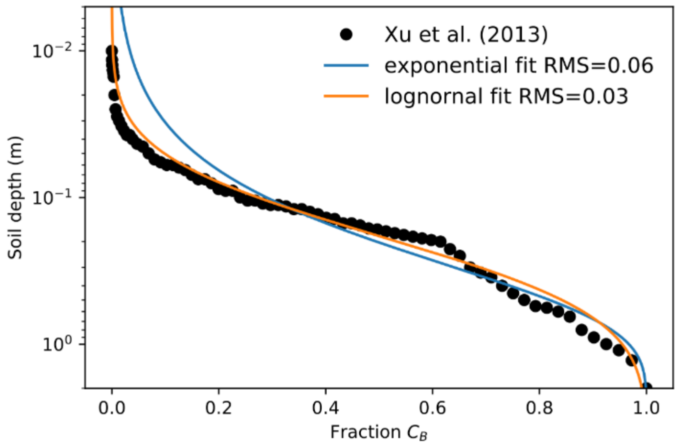

Supplementary Figure 1 | Decay of bacterial biomass carbon (C_B) with soil depth. The cumulative fraction of C_B taken from Xu *et al.* [7] is shown for a maximum depth of two meters (black symbols). The exponential model as reported by Xu *et al.* was fitted (blue line). A lognormal fit (orange line) shows an overall better alignment with the data, especially in the upper 10 cm. The root mean square errors (RMS) are reported in the legend.

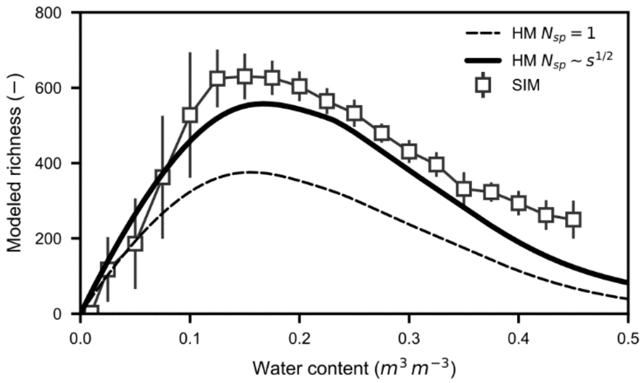

Supplementary Figure 2 | Comparison of the heuristic model (HM) with the spatially-explicit individual-based model (SIM) on surfaces (two dimensional domains). Square symbols and bars (mean ± SD, n = 12) depict richness predicted by the SIM rarified to 1000 counts. The aqueous-phase fragmentation-based HM (solid line) captures the trend in simulated richness with water content (m^3 m^{-3}). The proportionality of the number of species per habitat N_{sp} to the domain's dimensionality (surface or volume) and to the size *s* of the aqueous habitats (N_{sp}~$s^{1/2}$) may explain the difference between SIM and the single species HM (dashed line).

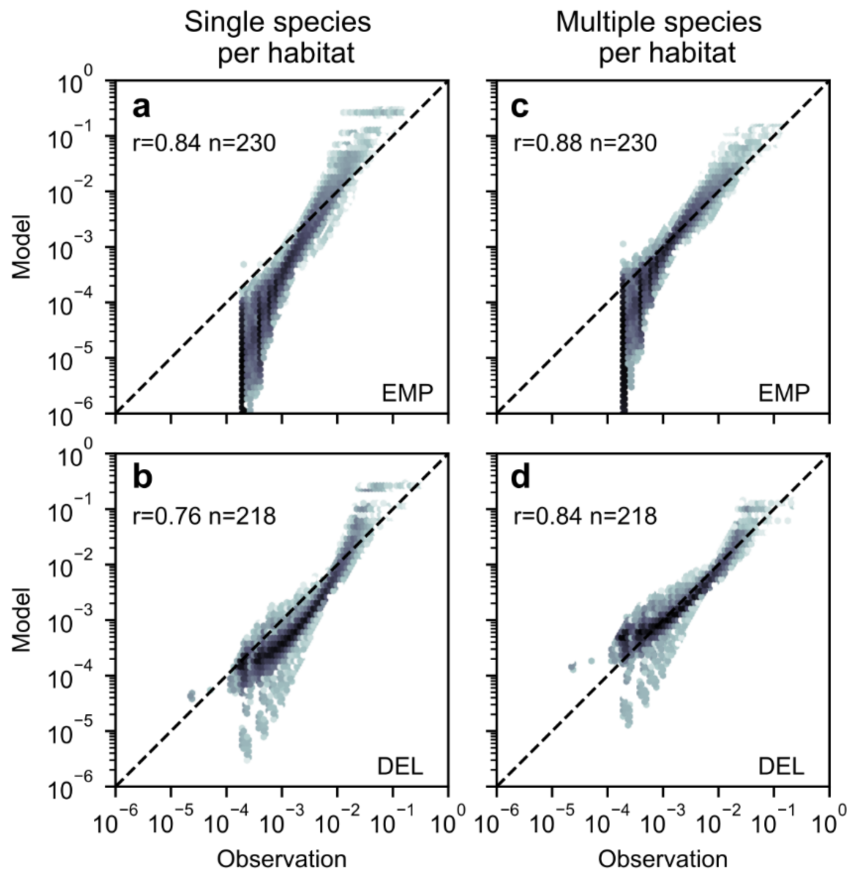

Supplementary Figure 3 | Modeled and observed soil bacterial species abundance distributions (SAD). Comparison of relative abundances from empirical observations (x-axis) with estimates of the aqueous-phase fragmentation-based heuristic model (HM; y-axis). Scenarios with single and multiple species per aqueous habitat are compared to observations. A 1:1 line and Pearson correlations are shown for both soil bacterial diversity datasets. **a**, Relative SADs from the Earth Microbiome Project (EMP) and **b,** from a recent study by Delgado *et al.* (DEL) considering a single species per habitat. The consideration of multiple species per habitat with the number of species N_{sp} proportional to the dimensionality and size s of the habitat ($N_{sp}{\sim}s^{1/3}$) improves the agreement with model predictions for both datasets; **c,** EMP and **d,** DEL, respectively.

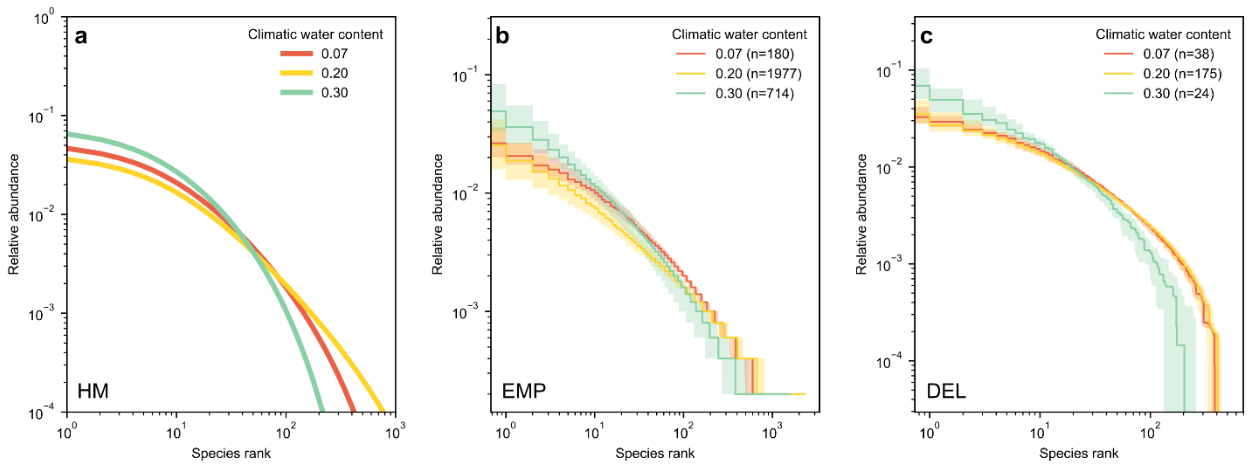

Supplementary Figure 4 | Qualitative comparison of theoretical and empirical species abundance distributions (SADs) for three climatic water contents (m^3 m^{-3}). **a,** SADs generated for median carrying capacity using the aqueous-phase fragmentation-based heuristic model (HM). Empirically observed SADs are grouped into equally spaced intervals of climatic water content (midpoint in legends) for **b,** the Earth Microbiome Project (EMP)[5] data and **c,** for a recent study by Delgado *et al.* (DEL)[6]. For each group the median (solid line) and the interquartile range (shading) as well as the number of samples are reported.

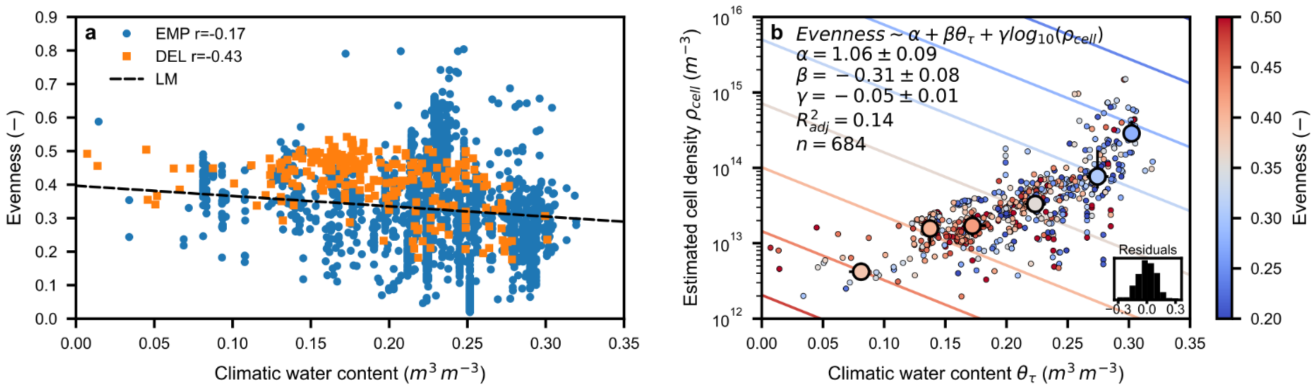

Supplementary Figure 5 | Empirically observed trends of soil bacterial evenness. **a**, Decrease of bacterial community evenness with climatic water content. Pearson correlation *r* for individual samples of both diversity datasets (EMP[5] n = 2871, DEL[6] n = 237) are indicated in the legend. The trend line shows a linear model (LM, see **b**) evaluated for median cell densities. **b**, The linear model was fitted to the empirical data for all sampled locations (n = 684) and the response surface of evenness is shown as a function of climatic water contents and cell densities (colored contours). Samples with cell densities lower than 10^{12} m^{-3} were removed prior to fitting the model as indicated in the figure. Negative slopes (β, γ) suggest that evenness is jointly reduced by increasing climatic water contents and cell density. Model residuals are not indicative of a persistent bias. Additionally, evenness is shown for bins of water contents (median ± IQR) to highlight the central tendency.

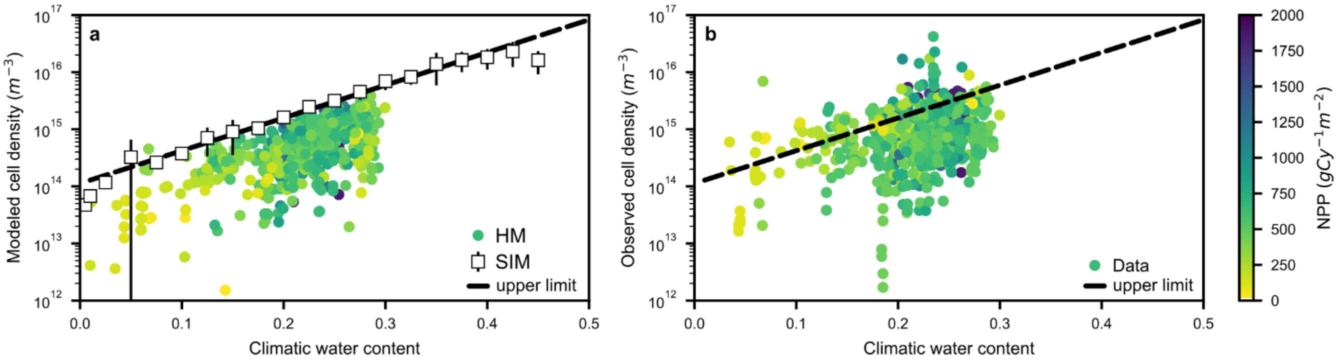

Supplementary Figure 6 | Relation of cell density with climatic water content (m^3 m^{-3}) based on net primary productivity (NPP). **a,** Modeled cell density as a function of NPP, mean annual temperature and soil depth using the heuristic model (HM; colored circles). The dashed regression line represents the mean tendency of the HM when integrated over the entire soil profile of 1 m and thus provides a theoretical upper bound. The dependency on climatic water contents was not explicitly modeled, it is rather indicative of the NPP's relation with hydration regime. The open black symbols are results of the spatially-explicit individual based model (SIM; mean ± SD, n = 12) where enough carbon to support a cell density of 10^{17} m^{-3} was prescribed. The dependency on water contents results from spatial variations in nutrient fluxes with water contents. **b,** Observed values of cell densities[7] are bounded by the theoretical upper limit.

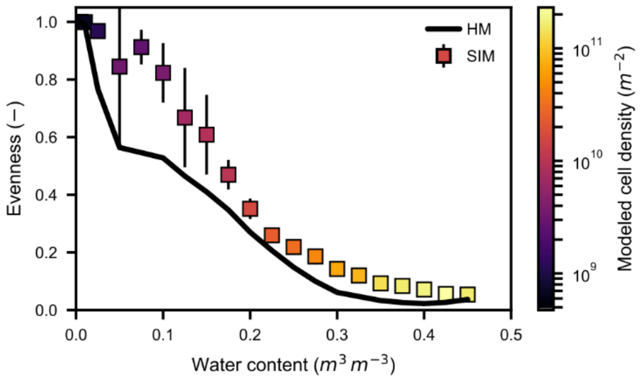

Supplementary Figure 7 | Comparison of evenness estimated using the aqueous-phase fragmentation-based heuristic model (HM) and the spatially-explicit individual-based model (SIM) for different water contents and carrying capacity. The HM (solid line) is evaluated in two dimensions for every value pair of modeled cell density and water contents obtained from the SIM (square symbols and bars – mean ± SD, n = 12). Colors indicate modelled cell density from the SIM.

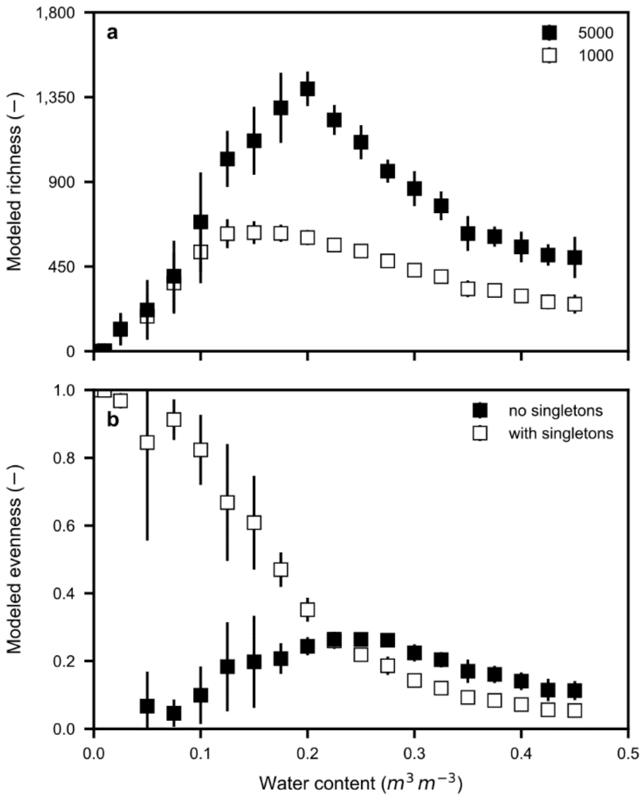

Supplementary Figure 8 | Pre-processing and sampling of simulated bacterial species abundance data may exert a strong effect on the deduced diversity metrics. **a** and **b**, Simulated soil bacterial diversity metrics using the spatially explicit individual-based model (SIM, mean ± SD, n = 12 different simulations). **a,** Rarefying to 5000 counts leads to higher magnitudes (compared to 1000 counts) of observed bacterial richness, yet the trends with water content remain consistent. **b**, Removing singletons (species sampled only once) exerts a strong influence on bacterial community evenness. This pre-processing step could distort the apparent relation of bacterial community evenness with climatic water content.

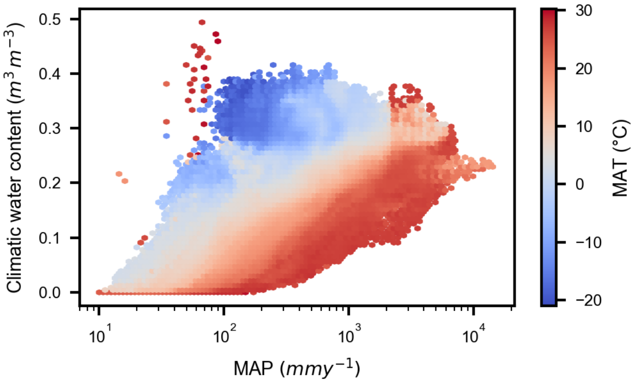

Supplementary Figure 9 | Global distribution of climatic soil water contents in relation with mean annual precipitation (MAP) and mean annual temperature (MAT). MAT spans climatic regions with different potential evapotranspiration (or aridity) and distinguishes locations (together with soil type) where climatic water contents may vary for the same MAP. For example, colder regions tend to require less MAP to attain relatively high climatic soil water contents.

References

1. Beck, H. E. *et al.* MSWEP V2 Global 3-Hourly 0.1° Precipitation: Methodology and Quantitative Assessment. *Bull. Amer. Meteor. Soc.* **100**, 473–500 (2019).
2. Fick, S. E. & Hijmans, R. J. WorldClim 2: new 1-km spatial resolution climate surfaces for global land areas: new climate surfaces for global land areas. *International Journal of Climatology* **37**, 4302–4315 (2017).
3. Hengl, T. *et al.* SoilGrids250m: Global gridded soil information based on machine learning. *PloS one* **12**, e0169748 (2017).
4. Zhao, M., Heinsch, F. A., Nemani, R. R. & Running, S. W. Improvements of the MODIS terrestrial gross and net primary production global data set. *Remote Sensing of Environment* **95**, 164–176 (2005).
5. Thompson, L. R. *et al.* A communal catalogue reveals Earth's multiscale microbial diversity. *Nature* **551**, 457–463 (2017).
6. Delgado-Baquerizo, M. *et al.* A global atlas of the dominant bacteria found in soil. *Science* **359**, 320–325 (2018).
7. Xu, X., Thornton, P. E. & Post, W. M. A global analysis of soil microbial biomass carbon, nitrogen and phosphorus in terrestrial ecosystems. *Global Ecology and Biogeography* **22**, 737–749 (2013).
